# The Study of Egg Allergy in Children With Atopic Dermatitis

**DOI:** 10.1097/WOX.0b013e3181abe7cb

**Published:** 2009-07-15

**Authors:** Tahmineh Salehi, Zahra Pourpak, Shahnaz Karkon, Raheleh Shokouhi Shoormasti, Samineh Kamali Sabzevari, Masoud Movahedi, Mohammad Gharagozlou, Mostafa Moin

**Affiliations:** 1 Immunology, Asthma and Allergy Research Institute, Tehran University of Medical Sciences, Tehran, Iran; 2 Department of Immunology, Children's Medical Center, Tehran University of Medical Sciences, Tehran, Iran

**Keywords:** egg allergy, atopic dermatitis, food allergy, specific IgE cutoff point

## Abstract

**Context**

Food allergy plays a significant pathogenic role among children with atopic dermatitis (AD).

**Objective**

The aims of this study were to evaluate allergy to egg in these children and determine the egg specific immunoglobulin E (IgE) cutoff point.

**Design and Setting**

It was a cross-sectional study that took place at Immunology, Asthma and Allergy Research Institute from 2005 to 2007.

**Methods**

Children younger than 14 years old with AD entered the study. Careful medical histories were taken and skin prick and Immuno-CAP tests with the most commonly offending foods (cow's milk, egg, wheat, peanut, and soy) were performed. Children with a clear, positive history of food allergy and a positive IgE-mediated test or those with positive responses to both IgE-mediated tests were determined to have food allergies. The egg-specific IgE level cutoff point was determined.

**Results**

A hundred patients entered the study (from 2 months to 12 years old). They were divided into 3 age groups: first <2 years, second from 2 to <6 years, and third from 6 to 14 years. The most common food allergens were egg (39.22%) in the first, cow's milk (35.13%) and egg (32.43%) in the second, and peanut (25%) and egg (16.67%) in the third group. The egg-specific IgE cutoff point value was 0.62 kUA/L (kilounits of allergen-specific IgE per liter). The positive predictive value was 95%.

**Conclusion**

Prevalence of egg allergy is highly significant in patients with AD.To use egg-specific IgE level cutoff point, the patient population under study must be considered.

## 

A topic dermatitis (AD) is a chronic and relapsing inflammatory cutaneous disease usually beginning at infancy, which is frequently associated with a family history of allergic diseases and often occurs in children with a personal history of other atopic disorders.[1-4] It is said to be the most common inflammatory skin disease in childhood;[5] it develops on dry skin with a hallmark of intense pruritus[2] and is associated with immunoglobulin E (IgE)-mediated sensitization in the majority of patients[2].

AD presents as a major public health problem[2] with a significant increase in prevalence in industrialized countries during the past 3 decades[1]. It affects 0.5% to 1% of the population[6] and 2% to 10% of adults but its prevalence in children has been reported from 5% to 30%.[1,7,8]

Development of atopic disease seems to depend on both genetic and several environmental factors[8]. More prevalence of AD in urban areas is attributed to better hygienic conditions, which prevents children from being exposed to infections at an early age and increases the susceptibility to allergic diseases.[1,9] Increased exposure to various kinds of pollutants and indoor allergens and a decline in breastfeeding, and also more awareness of atopic dermatitis, have been suggested as reasons for the increased frequency of this disease[10].

For unknown reasons, in more than 60% of cases AD may enter into complete remission during puberty[2,9] and up to 70% before adolescence[1]. In one study of 192 children with AD, 43.2% of them were in complete remission after their second birthday[5].

Food allergy (hypersensitivity) refers to an abnormal immunologic response, an adverse reaction attributed to the exposure to a specific food that is not related to any physiological effect of that food[11]. Approximately 6% of young children and 3.7% of adults in the United States have a food allergy[12]. The diagnosis requires skillful history taking, which is confirmed by either skin prick testing or demonstration of specific IgE antibody to the food by in vitro methods (eg, ImmunoCap, Pharmacia, Uppsala, Sweden)[13].

Several studies from the 1920s have noted the pathogenic role of food allergy in AD,[6,14] which is said to complicate the treatment of AD in 40% of these children.[15,16] The overall prevalence of food allergy is estimated at about 30%[17,18] in AD. But because of the nonspecific symptoms, early diagnosis of patients with food allergies is often missed[19].

Among food allergies, egg allergy is very common, affecting 1% to 2% of young children,[20] predominantly preschool-age children[21]. Egg-allergic reactions are commonly first observed in the second 6 months of life[22]. It is second overall only to milk allergy in prevalence and in most studies has been shown to be the most common food allergy in children with atopic dermatitis.[20,23]

In one study of 107 infants with AD and no known egg ingestion, 67% had evidence of IgE sensitivity to egg and positive reactions to an oral food challenge[22].

A majority of patients with egg allergy will develop egg tolerance[20]; about 20% of them will outgrow their egg allergy in the first 12 months[22] and approximately two thirds of them by 5 years old[21] (in contrast to peanut allergy, which usually persists into older childhood and adult life[22]). The prevalence of IgE-mediated reactions to egg in a population-based study of 2 year olds was 1.6%. However, at 7 years old only 0.2% of children had a positive SPT to egg[21].

Several studies have been done to validate the cutoff point for food-specific IgE levels to predict the presence of food allergy to make a diagnosis, reducing the need for performing diagnostic food challenge testing.[24-26]These studies support the idea that quantifying serum-specific IgE antibodies is useful for predicting the probability of suffering from an allergic disease[25].

The aim of this study was to evaluate the prevalence of egg allergy as an associating factor in children with atopic dermatitis, compared with other common food allergens, and to specify the egg-specific IgE cutoff point separating patients with egg allergy from those without egg allergy.

## Methods

This cross-sectional study was performed on children diagnosed with AD who were referred to the Allergy and Immunology Clinic at Children's Hospital Medical Center from 2005 to 2007.

All patients fulfilled the criteria of Hanifin and Rajka in at least 3 major and 3 minor criteria[6,7,10] for the diagnosis of atopic dermatitis. To diagnose food allergy, a careful, detailed medical history was taken and patients were questioned about their complaints and other allergic symptoms caused by specific food allergens.

Skin prick tests (SPTs) with whole egg and 4 of the most commonly offending food allergens in children including cow's milk, wheat, peanut, and soy[18,27,28] were performed on 84 patients; the reagents were prepared from Dome/Hollister-Stier Laboratories, Canada. The SPTs were performed with normal saline as a negative control and 1 mg/mL histamine as a positive control. Those who did not have any responses to the negative control test and had a positive response to histamine (wheal diameter more than 5 mm) were tested with allergens. A wheal diameter more than 3 mm (corresponding to a wheal area of 7 mm^2^) and flare diameter more than 10 mm were determined as positive tests[29].

The levels of food allergen-specific serum IgE were determined using a CAP System fluoroscein-enzyme immunoassay (CAP) [limit of the assay was less than 0.35 kUA/L (kUA/L, kilounits of allergen-specific IgE per liter)] (Pharmacia Diagnostics AB, Uppsala, Sweden) for 55 patients--who were younger than 2 years old, had diffuse dermatological involvement, were not cooperative, or were taking antihistamines or topical steroids--and were evaluated on a 0-5 scale (we reported ≥2+ response of the tests)[10]. The analysis of the receiver operating characteristic curves was done by SPSS for Windows (version 14) to determine the egg-specific IgE level cutoff point.

Also, total serum IgE with ELISA kits (Diagnostic-USA) was measured for 69 patients.

Children with a positive history of food allergy (a positive history of eczema exacerbation in the first 24 hours after the addition of one specific food to the patient's diet and the improvement of eczema after the 2-week elimination of that food from the diet) and a positive IgE-mediated test (SPT or ImmunoCAP) or those with positive responses in both IgE-mediated tests to one specific food allergen were determined as patients with allergy to that food.[11,30,31]

## Results

One hundred patients with atopic dermatitis (from 2 months to 12 years old) participated in this investigation. The mean and median ages at time of diagnosis were 33.31 and 23 months. A positive family history of allergic disease was identified in 81.63% (80/98) of the children. About half (51%) of the patients with AD were younger than 2 years old and 27 (27%) were equal to or younger than 1 year old, whereas in the group of patients with egg allergy 20 (60.6%) of them were younger than 2 years old and 13 (39.4%) were in their first year of life.

Skin prick and CAP tests were positive for egg allergen in 36.36% (28/77) and 41.81% (23/55) of the patients, respectively.

A careful, detailed medical history and IgE-mediated tests (ImmunoCAP and/or SPT) revealed that 34 AD patients (34%) had egg allergy, 25 (25%) milk allergy, 13 (13%) wheat allergy, 12 (12%) peanut allergy, and 9 (9%) soy allergy.

Although 36.36% of the patients had positive SPTs and 41.81% had positive CAP tests for egg allergen (egg sensitivity), the prevalence of egg allergy was 34%.

Patients were divided into 3 groups according to age: the first group included patients younger than 2 years (51 patients), the second between 2 and 6 years (37 patients), and the third group between 6 and 14 years (12 patients). The relationship of age with the presence of egg allergy is shown in Table 1.

**Table 1:** The Age Relationship With Egg Allergy in Patients With Atopic Dermatitis

	**Results**		
	**With Egg Allergy**	**Without Egg Allergy**	**Total**
Age group 1	20	31	51
0-23 months	39.22%	60.78%	100%
Age group 2	12	25	37
24-71 months	32.43%	67.57%	100%
Age group 3	2	10	12
≥ 72 months	16.67%	83.33%	100%
Total	34	66	100
	34%	66%	100%

The most common food allergens in the first group, from greatest to least, were egg (39.22%), cow's milk (21.57%), wheat (17.65%), soy (7.84%), and peanut (5.88%).

In the second group, the first and second most common allergens were cow's milk (35.13%) and egg (32.43%), and the other allergens ranked as follows: peanut (16.22%), wheat (10.81%), and soy (10.81%).

In the third group, peanut was the most common food allergen (25%) and the others ranked as follows: egg (16.67%), cow's milk (8.33%), soy (8.33%), and wheat (0%).

Twenty-nine percent of the patients were female and 71% were male. The male-to-female ratio in patients with egg allergy was 2.09 and that in patients without egg allergy was 2.67; the difference was not significant (*P *= 0.596).

**Figure 1:**
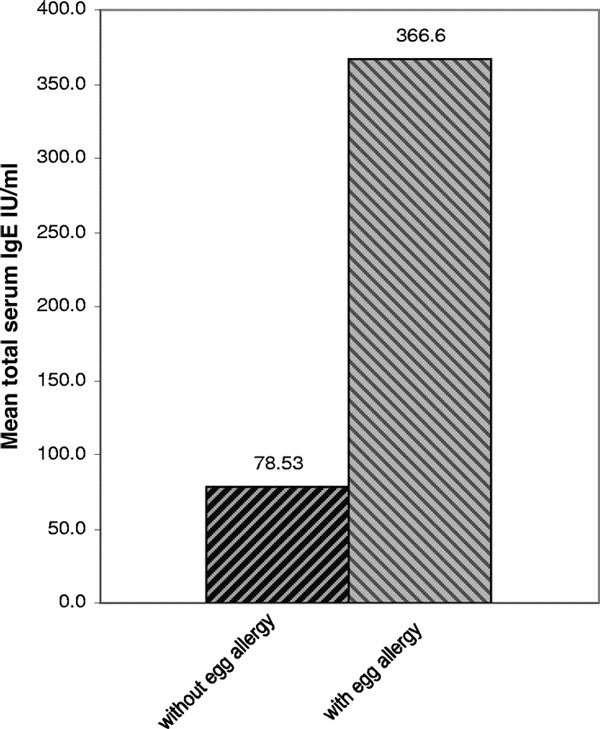
**Comparison between total serum IgE in patients with AD and with or without egg allergy**.

Total serum IgE levels in children with egg allergy compared with children without egg allergy are shown in Figure 1. Mean total IgE serum in these groups was 366.6 IU/mL and 78.53 IU/mL, respectively (*P *= 0.01). Mean and median total IgE in all AD children were 182.9 IU/mL and 23.5 IU/mL.

In the group of patients with egg allergy, 17 (50%) patients were allergic to milk, 12 (35.29%) to wheat, 6 (17.64%) to peanut, and 4 (11.76%) to soy. In children with egg allergy, 12 (35.29%) did not have any other food allergies, compared with 50 (75.76%) in children without egg allergy. The presence of other food allergies in children with egg allergy and children without egg allergy is shown in Table 2. (The overlap of allergies to different foods should be considered in both groups--patients with egg allergy and those without egg allergy).

**Table 2:** Concomitant Food Allergies in Children With Atopic Dermatitis, Comparing Patients With Egg Allergy to Those Without Egg Allergy

	**Patients With AD and Egg Allergy** **(Total = 34)**	**Patients With AD and Without Egg Allergy** **(Total = 66)**	***P***
Milk allergy	17 (50%)	8 (12.12%)	<0.001
Wheat allergy	12 (35.29%)	1 (1.51%)	<0.001
Peanut allergy	6 (17.64%)	6 (9.09%)	0.020
Soy allergy	4 (11.76%)	5 (7.57%)	0.106
None	12 (35.29%)	50 (75.76%)	<0.001

The mean of the egg CAP score (0-5) in children with AD and egg allergy was 2.64. It was 2.71 in the first age group (0-23 months), 2.5 in the second group (24-71 months), and 2.5 in the third group (≥72 months).

The egg-specific IgE cutoff point value was 0.62 kUA/L with 100% sensitivity and 96.7% specificity with a positive predictive value of 95%.

## Discussion

The diagnosis of food allergy is indicated by clinical history. Food-specific IgE antibodies, found in serum or as a result of a SPT, support the existence of allergy to that specific food, whereas the gold standard test for a definite diagnosis is a double-blind placebo-controlled food challenge (DBPCFC) test[25].

The number of patients who were considered to have positive egg allergy was 34 (34%), which was similar to that found in other studies[32]. Regarding, it is said that the role of food as a trigger in all infants with moderately severe to severe AD should be considered as well[33].

As results showed, a positive family history of allergy was 82%; this study confirms the finding that genetic predisposition to atopy plays a significant role in the development of AD[34]. So in families with a child who has AD and with a history of atopy, it would be beneficial to consider the presence of egg allergy. Also, most individuals who have food allergy have a family history of allergy too[13].

In this study the male-to-female ratio in AD patients was approximately 2.45, which is almost the same as that found in the studies by Pourpak et al and Bellioni-Businco et al on cow's milk allergy.[34,35] Also, in asthmatic patients it was shown that boys are affected more than 2 times often than girls,[36] but other studies are needed to prove a relationship between sex-dependent genes and egg allergy.

In our survey, 26% of children with AD were younger than 1 year, and in the group of patients with AD and egg allergy, only 13 (38.23%) were in the first year of life; however, it has been said that both IgE-mediated food allergy and AD have their greatest prevalence in the first 12 months of life[12].

As Table 1 shows, it seems that people with egg allergy will outgrow it with age and there is a significant difference among age groups in the prevalence of egg allergy. Also, by 6 years of age and older, egg allergy seems to resolve itself, as does cow's milk allergy,[20,22] in contrast to peanut allergy,[22] which usually persists into older childhood and adult life[18].

The relationship between the presence of egg allergy and total serum IgE was another entity to be confirmed. The difference between total serum IgE in children with egg allergy and that in children without egg allergy was significant (*P *< 0.01). As the results show, in patients with Atopic Dermatitis and egg allergy, the mean total serum IgE is significantly higher than that of patients who do not have egg allergy (366.6 vs 78.53 IU/mL). It shows that egg allergy may enhance the total serum IgE in patients with AD and egg allergy compared to patients with AD and other common food allergies; other possible causal factors such as *Staphylococcus aureus *toxin exposures need to be studied.

As mentioned, the egg-specific IgE cutoff point value in our study was 0.62 kUA/L, which was close to Boyano Martinez et al's result for egg white (0.43 kUA/L)[25].

Our finding was different from the CAP value found by Sampson and Ho (6 kUA/L for egg white), which was accompanied by a positive challenge test in more than 95% of their cases[37]. Also, Roehr et al reported 17.5 kUA/L for hen egg,[16] Osterballe and Bindslev-Jensen determined 1.5 kUA/L for egg white,[38] Celik-Bilgili et al reported 12.6 kUA/L,[39] and Komata et al found 25.5 kUA/L[40] for hen egg as the cutoff point.

This difference could be because of the different populations studied. The median age of our patients was 1.92 years (23 months), compared with 5.2 years in Sampson and Ho's study,[37] 16 months in Boyano Martinez et al's,[25] 13 months in Roehr et al's,[16] 2.2 years in Osterballe and Bindslev-Jensen's,[38] 13 months in Celik-Bilgili et al's,[39] and 1.3 years in Komata et al's[40]. Also, the patient selection criteria in each study were different. Sampson and Ho (United States) studied a group of patients with food allergy who were highly atopic and all of them had atopic dermatitis[37]. Boyano Martinez et al (Spain) included children younger than 2 years who were suspected of having egg allergy[25]. Roehr et al (Germany) worked with children who had AD[16]. Osterballe and Bindslev-Jensen (Denmark) studied children with AD and suspected egg allergy[38]. Celik-Bilgili et al (Germany) included children with suspected food allergy[39]. Komata et al (Japan) recruited children suspected of suffering from egg and milk allergies[40]. In contrast, we observed all children with AD referred to our allergy clinic--whether or not they were suffering from another type of allergy. Our criteria were almost the same as those used by Roehr et al[16].

Next, the method of food allergy diagnosis differed in each study. We did not perform a DBPCFC test for the diagnosis of food allergy because of our limitations. Patients who revealed a history of food allergy and a positive response to one of the IgE-mediated tests (SPT and/or ImmunoCAP) or those with positive responses in both IgE-mediated tests to one or more food allergens were determined as having food allergy.[11,29,31] In contrast, in other studies the food challenge test was performed for a definite diagnosis.[16,25,37-40]

The specific IgE level predicting egg allergy varied from 0.35 kUA/L to more than 25 kUA/L in different published data. Therefore, our study admits that because of the great impact of the population studied and the inclusion criteria, results obtained in one population cannot be readily transferred to other ones.

Our results, which were similar to those from other studies,[20,27] revealed that the prevalence of egg allergy is highly significant in children with AD, and before the age of 2 years, it is even more common than cow's milk allergy. So an evaluation for food allergy and especially egg allergy should be considered in children with AD to advise relevant elimination diets.

Another concept to be mentioned is the concomitance of other food allergies in patients with egg allergy and AD. As the results show, cow's milk and wheat allergies are significantly prevalent in children with AD and egg allergy, compared with those children without egg allergy. Further studies are needed to confirm whether children with egg allergy and AD are more susceptible to other food allergies. As half (50%) of the patients with egg allergy and AD had milk allergy too, it is recommended that other surveys be carried out to establish a relationship between these 2 food allergens in children with AD.
